# Characteristics of Fine Particles in an Urban Atmosphere—Relationships with Meteorological Parameters and Trace Gases

**DOI:** 10.3390/ijerph13080807

**Published:** 2016-08-10

**Authors:** Tianhao Zhang, Zhongmin Zhu, Wei Gong, Hao Xiang, Ruimin Fang

**Affiliations:** 1 State Key Laboratory of Information Engineering in Surveying, Mapping and Remote Sensing, Wuhan University, Wuhan 430079, China; tianhaozhang@whu.edu.cn (T.Z.); weigong@whu.edu.cn (W.G.); fangrm@whu.edu.cn (R.F.); 2 College Information Science and Engineering, Wuchang Shouyi University, Wuhan 430064, China; 3 Collaborative Innovation Center for Geospatial Technology, Wuhan 430079, China; 4 School of Public Health, Wuhan University, Wuhan 430071, China; xianghao@whu.edu.cn

**Keywords:** fine particles, meteorological parameters, trace gases, “repeated, short-lived” nucleation events, central China

## Abstract

Atmospheric fine particles (diameter < 1 μm) attract a growing global health concern and have increased in urban areas that have a strong link to nucleation, traffic emissions, and industrial emissions. To reveal the characteristics of fine particles in an industrial city of a developing country, two-year measurements of particle number size distribution (15.1 nm–661 nm), meteorological parameters, and trace gases were made in the city of Wuhan located in central China from June 2012 to May 2014. The annual average particle number concentrations in the nucleation mode (15.1 nm–30 nm), Aitken mode (30 nm–100 nm), and accumulation mode (100 nm–661 nm) reached 4923 cm^−3^, 12193 cm^−3^ and 4801 cm^−3^, respectively. Based on Pearson coefficients between particle number concentrations and meteorological parameters, precipitation and temperature both had significantly negative relationships with particle number concentrations, whereas atmospheric pressure was positively correlated with the particle number concentrations. The diurnal variation of number concentration in nucleation mode particles correlated closely with photochemical processes in all four seasons. At the same time, distinct growth of particles from nucleation mode to Aitken mode was only found in spring, summer, and autumn. The two peaks of Aitken mode and accumulation mode particles in morning and evening corresponded obviously to traffic exhaust emissions peaks. A phenomenon of “repeated, short-lived” nucleation events have been created to explain the durability of high particle concentrations, which was instigated by exogenous pollutants, during winter in a case analysis of Wuhan. Measurements of hourly trace gases and segmental meteorological factors were applied as proxies for complex chemical reactions and dense industrial activities. The results of this study offer reasonable estimations of particle impacts and provide references for emissions control strategies in industrial cities of developing countries.

## 1. Introduction

Atmospheric aerosol particles not only greatly influence the global radiation budget and climate change by their associated radiative effects [1,2,3,4], but also demonstrate an association with respiratory and cardiovascular morbidity and mortality through a series of toxicological and epidemiological studies [5,6,7,8,9]. Existing standards for air quality, such as particulate matter with diameter smaller than 10 μm (PM_10_) and particulate matter with diameter smaller than 2.5 μm (PM_2.5_), are formulated based on the mass concentration of these particles, which means that the mass concentration of fine particles with aerodynamic diameters smaller than 2.5 μm are approximately neglected [10]. However, ultrafine particles (diameter < 0.1 μm), which are primarily affected by nucleation [11], normally make up a significant component of particle number concentration in many urban areas [12,13,14]. Moreover, various studies proved that the number concentration of ultrafine particles is more effective than the mass concentration of particles for predicting adverse health effects [15,16,17,18]. As ultrafine particles actually play a role in carrying adsorbed carcinogenic substances, the World Health Organization (WHO) has upgraded diesel exhaust from “probably carcinogenic” to “carcinogenic to humans” [19], because ultrafine particles are partly formed by vehicle exhaust and industrial emissions [20]. Thus, it is of great significance to measure particle size distribution (PSD) to fully reveal their environmental and health effects [10,21].

Extensive studies on PSD have been conducted in many European and American cities, which revealed that diurnal variations in PSD were linked closely with traffic-related emissions [22,23,24,25,26], and seasonal patterns of PSD included higher concentrations in winter and lower concentrations in summer [23,25]. As traffic emissions were considered widely as a main component of particles in an urban atmosphere, [27] divided traffic-related emissions into those from vehicle exhaust, which included studies of vehicle operating modes [28,29] and fuel types [30,31,32,33], and those from vehicle non-exhaust sources, such as from brake wear [34,35], tire-road surface interaction [34], and resuspension [20,34]. Industrial combustion emissions also contribute greatly to the ultrafine and fine particle fractions [27]. In addition, studies of growth processes in long-range transported aerosols have been reported by [36,37,38], and regional nucleation events, which were attributed to the ambiguous relationship between fine particles and black carbon (BC), were presented by [39,40,41].

Meteorological parameters, including temperature, relative humidity (RH), precipitation, wind speed, and planetary boundary layer height, appreciably influence PSD. Paatero [42] reported a fitting model between particle number concentration and meteorological variables in five European cities. The effects of meteorological parameters on PSD changed in conjunction with seasonal variation [43]. Moreover, the contribution of meteorological processes to PSD was demonstrated in the urban area of Helsinki, Finland [44].

As mentioned above, numerous studies on PSD were performed in developed countries. Meanwhile, China is also a large source region for anthropogenic aerosols in the world. Measurements of PSD in China have focused mainly on the Beijing-Tianjin-Hebei area, Yangtze River Delta region, Pearl River Delta region, and northwestern China [10,43,45]; however, analysis of PSD in central China remains limited, and there no studies existing in the literature about scientific research of aerosol particle size distribution or nucleation burst events in Wuhan.

This study obtained two years of number concentration data of particles with diameters ranging from 15.1 nm to 661.2 nm from the city of Wuhan since June 2012. The diurnal, monthly, and seasonal variations of PSDs and concentrations were analysed. In addition, the variation characteristics in temperature, RH, atmospheric pressure (AP), precipitation, wind speed, and mixing height and their relationships with particle concentrations in the nucleation mode, Aitken mode, and accumulation mode were investigated in detail. Moreover, a case analysis of a nucleation burst event in winter was conducted using air pollutants (NO_2_, CO, SO_2_, and O_3_), BC, and meteorological parameters to explore the key factors causing the durability of high particle number concentrations during winter in Wuhan.

## 2. Materials and Methods

### 2.1. Sampling Location

**Figure 1:**
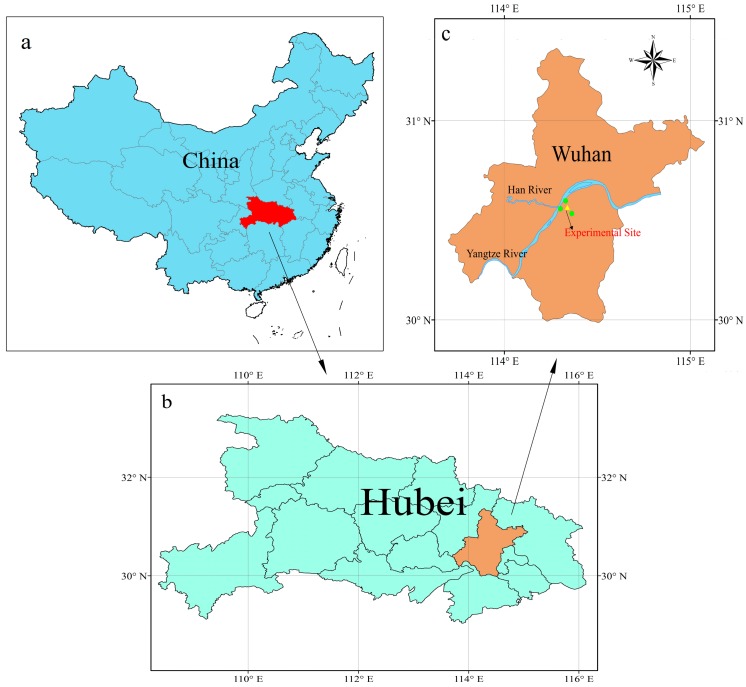
Geographic location of experimental area and sampling site: (**a**) location of Hubei Province in China; (**b**) location of Wuhan in Hubei Province; and (**c**) location of experimental site and three air quality monitoring stations in Wuhan.

The city of Wuhan in central China (Hubei Province) has experienced dense urbanization and industrial activities, with the production of steel from the Wuhan Iron and Steel Corporation ranking fifth and eighth in the world, respectively, in 2013 and 2014 [46]. Figure 1 shows the geographic location of Wuhan, which is situated at the confluence of the Yangtze and Han rivers, divided into three administrative regions, and characterized by a typical subtropical monsoon climate. The instruments for monitoring atmospheric particle size distribution and BC were installed in the attic of the State Key Laboratory of Information Engineering in Surveying, Mapping and Remote Sensing (Liesmars) at Wuhan University (30°53′ N, 114°38′ E). Hourly air pollutants, including NO_2_, CO, SO_2_, and O_3_, were measured at air quality monitoring sites supervised by the Ministry of Environmental Protection of the People’s Republic of China. Figure 1c shows these three air quality monitoring sites (green dots), which were selected to provide self-calibration data avoiding accidental error, around the experimental site. The straight-line distance between these three air quality monitoring sites and the experimental site is approximately five kilometers. Hourly meteorological data, including temperature, RH, AP, precipitation, and wind speed, in addition to mixing layer height every eight hours, were gleaned from a meteorological station in Wuhan, which is supervised by the China Meteorological Administration.

### 2.2. Measurements

#### 2.2.1. Particle Number Size Distributions

Continuous hourly number size distributions of particles between 15.1 nm to 661.2 nm in diameter were measured using a scanning mobility particle sizer (SMPS, model 3936L75-N, TSI Inc., Shoreview, MN, USA) from 1 June 2012 to 31 May 2014. The ambient air was sampled by the SMPS via an approximately 5 feet long and 0.5 inch wide stainless steel tube through the roof of the laboratory, which is about 50 feet above the ground in Wuhan University, with the sheath and sample flows kept stable at 3.0 L·min^−1^ and 0.3 L·min^−1^, respectively. A bipolar charger in an electrostatic classifier (EC, model 3080, TSI Inc., Shoreview, MN, USA) was used to charge the particles to a known charge distribution. These charged particles were classified by a differential mobility analyser according to their traverse ability in an electrical field, and a condensation particle counter (CPC, model 3775, TSI Inc., Shoreview, MN, USA) was used to count the number concentration of particles in a respective size range. To ensure the quality of data, the volume of n-butyl alcohol and operational parameters, including temperature, voltage, and flow rate, were checked daily, and the valve connector was cleared weekly. Furthermore, the equipment software corrected for the instrumental multi-charged effect and diffusional loss. Excluding missing data and problematic data, 676 integrated days of valid particle number concentrations were recorded from June 2012 to May 2014.

#### 2.2.2. Black Carbon and Trace Gases

The hourly mass concentration of BC was measured using an AethaLometer (model AE-31, Magee Scientific, Berkeley, CA, USA), based on the estimation of mass concentration of BC that accumulates on a 1.6 cm quartz fibre filter every two minutes. The raw data measured by the AethaLometer was divided into seven channels acquired by wavelengths of 350 nm, 470 nm, 521 nm, 590 nm, 660 nm, 880 nm, and 950 nm. This study only utilized measurements at 880 nm, as this wavelength is considered the standard channel to exclusively estimate BC concentration [46]. As an AethaLometer of this type is well-known to exhibit measurement artefacts that can result in reported BC mass concentrations twice as high as the true value, the operative behaviour of the AethaLometer, as well as the filter, the quartz filtration membrane, and the stability of the light source, were checked daily. Furthermore, trimestral calibration comprising blank measurements and flow rate calibration occurred to avert seasonal-dependent measurement artefacts.

The hourly mean concentrations of trace gases, comprising NO_2_, CO, SO_2_, and O_3_, measured at the three national air quality monitoring sites encircling the experimental site, were downloaded from the China National Environmental Monitoring Centre’s website (http://113.108.142.147:20035/-emcpublish/). The continuous monitoring systems for NO_2_, CO, SO_2_, and O_3_ were composed of the sampling unit, analytical device, data collection unit, and calibration instrument. CO was measured adopting the gas filter correlation infrared absorption method and the nondispersive infrared absorption method (API model 300A). O_3_, SO_2_, and NO_2_ were measured via the UV-spectrophotometry, ultraviolet fluorescence (API model 200A), and chemiluminescence (Advanced Pollution Instrumentation, Model 100A) methods, respectively [43]. Similar to the AethaLometer, there was a necessary daily check on these hourly data to avoid abnormal data in the calculations.

### 2.3. Data Analysis

PSD is characterized empirically by a multi-lognormal structure, usually divided into four main modes: the nucleation mode (*D_p_* < 30 nm), Aitken mode (30 nm < *D_p_* < 100 nm), accumulation mode (100 nm < *D_p_* < 1 μm), and coarse mode (*D_p_* > 1 μm) [27]. In this study, for calculating particle number concentrations in different size categories, the diameter range of 15.1 nm to 661.2 nm was divided into three modes (15.1 nm < *D_p_* < 30 nm, 30 nm < *D_p_* < 100 nm, and 100 nm < *D_p_* < 661.2 nm, respectively).

To demonstrate better the characteristics of particle number concentrations and their size distributions, raw data of number size distributions were fitted to a multi-lognormal distribution according to the function [47]:

(1) $$\frac{dN}{d\left(\log\left(D_{p}\right)\right)}=\sum_{i=1}^{n}\frac{N_{i}}{\sqrt{2\pi }\log\left(\sigma_{g,i}\right)}\exp\left[-\frac{{\left(\log\left(D_{p}\right)-\log\left({\bar{D}}_{pg,i}\right)\right)}^{2}}{2\log^{2}\left(\sigma_{g,i}\right)}\right],$$

where $D_{p}$ (nm) is the particle diameter, and *N_i_* (cm^−3^), $\sigma_{g,i}$ (nm^2^), and ${\bar{D}}_{pg,i}$ (nm) represent the total number concentration, geometric standard deviation, and geometrical mean diameter, respectively.

### 2.4. Pearson Correlation Analysis

The Pearson correlation coefficient is a number between −1 and 1 that measures the linear correlation between two variables, where 1 refers to total positive correlation and −1 refers to total negative correlation. It was developed by Karl Pearson [48] and was used as a measurement of the degree of linear dependence between particle number concentrations and meteorological parameters [49]. The calculation formulas can be expressed as follows:

(2) $$\rho =\frac{\sum_{i=1}^{n}\left(X_{i}-\bar{X}\right)\left(Y_{i}-\bar{Y}\right)}{\sqrt{\sum_{i=1}^{n}{\left(X_{i}-\bar{X}\right)}^{2}}\sqrt{\sum_{i=1}^{n}{\left(Y_{i}-\bar{Y}\right)}^{2}}},$$

where ρ is the Pearson correlation coefficient, and $\bar{X}$ and $\bar{Y}$ are the mean of variables *X* and *Y*.

## 3. Results and Discussion

### 3.1. Overview of Particle Number Concentrations

The particle number concentrations in different size segments were compared with those in other areas of the world, such as the United States, Europe, East Asia, and some regions in China. As Table 1 demonstrates, not only submicron particle total number concentrations but also particle number concentrations of each mode in Wuhan were relatively high compared with developed countries and similar to cities in China. The results that number concentrations of submicron particles in the size ranges of 15–30 nm, 30–100 nm, and 100–661 nm reached 4923 cm^−3^, 12,193 cm^−3^ and 4801 cm^−3^, respectively, are evidence that central China has been suffering severe atmospheric fine particle contamination. Although there is scant published literature about fine particle concentrations in industrial regions of developing countries, it is of vital importance to reveal such characteristics in Wuhan for providing reference to support future policy, due to its differing population, economic development level, and energy structure.

### 3.2. Meteorological Dependence of Particle Number Concentrations

**Figure 2:**
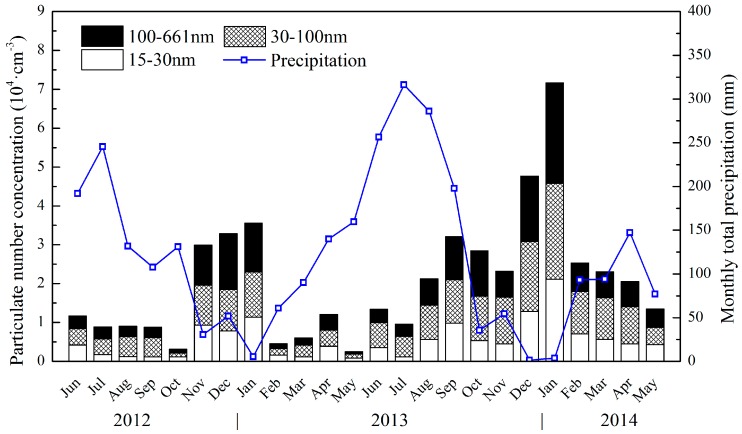
Monthly variation in particle number concentrations for the three modes and monthly total precipitation, where the blank, gridded, and **black** areas represent the nucleation, Aitken, and accumulation modes, respectively, and the **blue** hollow squares represent the monthly total precipitation.

As many studies have indicated that precipitation [56,57], wind speed [36,44], temperature [36], RH [56], and boundary layer structure [44,58] had different effects on particle number concentrations in various global regions, meteorological conditions are essential factors in analysing the levels and variations of particle number concentration. The relationships between precipitation and particle number concentrations in the three modes are shown in Figure 2. As influenced by typical subtropical monsoon climate, it rained most frequently in summer with an average of over 200 mm of precipitation per month, and least frequently in winter with an average below 50 mm per month, while all three modes had inverse models, obviously demonstrating the negative correlation between the particle number concentrations and precipitation in Wuhan. Furthermore, the fact that the proportion of the three modes remained proximate when the levels of particle number concentrations were relatively high with a lack of precipitation should not be ignored. However, when the particle number concentrations remained at a relatively low level, which was usually combined with abundant precipitation, the Aitken mode was the major constituent of the total number concentrations, and the proportion of the accumulation mode sharply decreased. Moreover, it also should not be neglected that when the monthly total precipitation was kept at a moderate level ranging approximately from 50 to 200 mm, primarily in spring and autumn, the particle number concentrations in three modes would have equivocal relationships with the precipitation. The possible reason mainly exists in the precipitation pattern that 75 mm precipitation twice in one month and 5 mm precipitation every day in one month would both contribute to 150 mm monthly total precipitation. Thus, precipitation at a moderate level in Wuhan is not linked distinctly with particle number concentrations.

**Figure 3:**
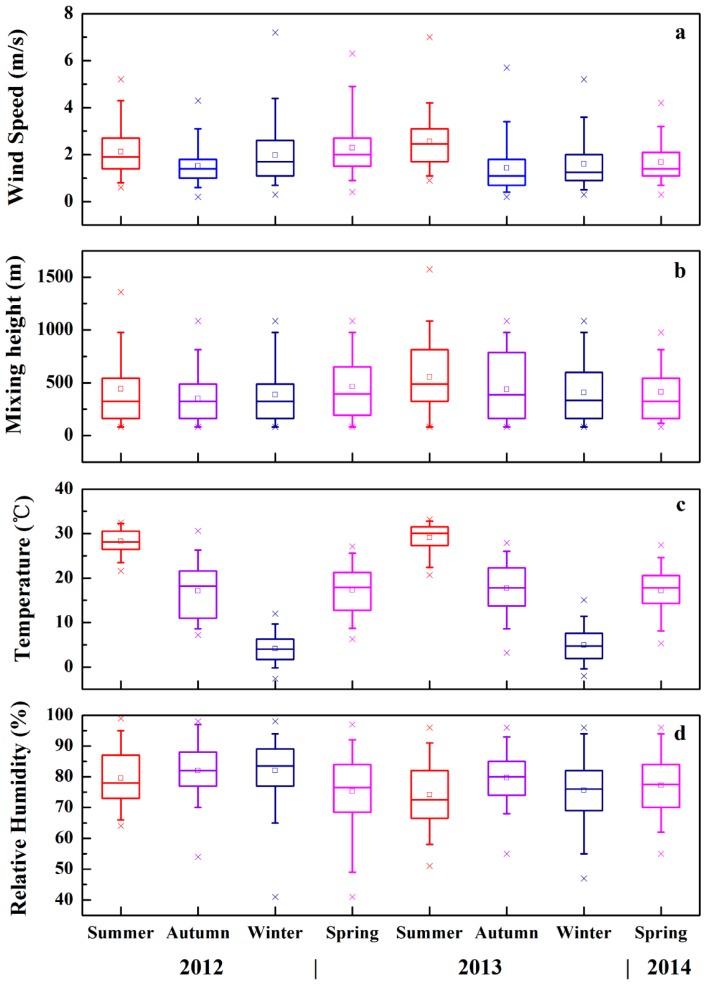
Seasonal variations in wind speed (**a**); mixing layer height (**b**); temperature (**c**); and relative humidity (**d**) in Wuhan during the two-year experiment. The upper and lower edges of the boxes represent the 25th and 75th percentiles; the horizontal line and square in the box show the median and mean values, respectively; the lower and upper whiskers of each box represent the 5th and 95th percentiles, respectively; and the lower and upper crosses represent the 1st and 99th percentiles, respectively.

Figure 3 shows the seasonal variation in wind speed, mixing layer height, temperature, and RH during the two-year experiment. There were obvious seasonal variation patterns for temperature and wind speed. The average temperature and wind speed in summer were approximately 29 °C and 2.2 m·s^−1^, respectively, which were clearly higher than they were in other seasons. Average RH in summer was lower than it was in other seasons, possibly caused by extremely high temperatures in summer, whereas the relatively higher RH in other seasons may have been affected by the geographic location, i.e., lying on the Yangtze River. Moreover, the mean value of mixing layer height was approximately 500 m, which was a little higher in summer and autumn, which is significantly lower than it was in Beijing, namely 1100 m in annual average [59] and Lanzhou, namely 1800 m averaged in summer and 800 m averaged in winter [43]. In addition, the correlation between the mixing layer height and temperature remained equivocal, indirectly indicating that the mixing layer height not only depends on temperature, but also is influenced by climatological and geographical factors [60].

Table 2 further demonstrated the correlation between the number concentrations of particles in different size bins and meteorological parameters both averaged by month, which was indicated by Pearson coefficients. The particle number concentrations in the three modes were calculated, first by fitting equations with precipitation, wind speed, RH, AP, temperature, and mixing layer height. Then, significance level, namely *p*-value, was assigned according to the frequency with which the Pearson coefficients generated from stochastically permuted data exceeded the original observed data.

As shown in Table 2, in the Wuhan urban area, there was a significant negative correlation between precipitation and particle number concentrations in the nucleation, Aitken, and accumulation modes, with Pearson coefficients of −0.505, −0.477, and −0.537 respectively. The Pearson coefficients for precipitation with particle number concentrations follow the pattern: accumulation mode > nucleation mode > Aitken mode. Moreover, the Pearson coefficients between particle number concentrations in the accumulation mode were significant with *p* < 0.01, which directly indicates an apparent washout effect of particles in the accumulation mode. Furthermore, although precipitation had diminished particle number concentrations in the nucleation and Aitken modes to some extent, the precipitation intensity may have been the key factor determining the scouring effect on the fine particles [61].

Similarly, number concentrations of particles in the nucleation, Aitken, and accumulation modes displayed a negative relationship with wind speed, with Pearson coefficients of −0.434, −0.390, and −0.408, respectively, indicating a dilution effect of the wind. However, the Pearson coefficient between wind speed and particle number concentrations in the Aitken mode was not significantly obvious with *p* > 0.05, and the correlation between wind speed and particle number concentrations seemed less than satisfactory, possibly caused by the reason that wind sometimes brought in exogenous pollutants rather than diluting the local atmosphere [46]. Likewise, particle number concentrations in the nucleation, Aitken, and accumulation modes demonstrated positive relationships with AP, with Pearson coefficients (*p* < 0.01) of 0.547, 0.544, and 0.579, respectively. It could be explained that low AP leads to convergence updraft, which promotes the dispersion of particles from near ground to the upper air, resulting in a reduction of particle number concentrations. In contrast, the downdraft caused by high pressure reinforces the downward movement of particles [62]. Reference [23] revealed that the annual variation of particle number concentrations in three modes was proportional inversely to temperature, which was almost identical to the situation in Wuhan, with Pearson coefficients (*p* < 0.05) of −0.57, −0.515, and −0.563.

However, there rarely existed a correlation between the number concentrations of particles in the three modes and RH. Figure 3d illustrates that RH in the Wuhan urban area was over 75% most of the time. On the one hand, RH plays an important role as a catalyst in hygroscopic growth of particles from primary and secondary emissions in the nucleation and Aitken modes. On the other hand, high RH in conjunction with an unstable atmosphere or intense convection may contribute to cloud condensation nuclei and wet deposition [63].

Similarly, the ambiguous relationship between particle number concentrations in the three modes and mixing layer height is shown in Figure 2 and Figure 3b, respectively. As mixing layer height is an important factor characterizing the potential of emitted air pollutant uptake [64], it indicates the capability of diffusion by the atmosphere. Identified by means of the turbulence parameters, temperature inversions and water vapour concentration, the mixing layer height could possibly change the vertical profile of aerosol number concentrations [65]. Therefore, along with the expansion and compression of the atmosphere, particle number concentrations may change accordingly, lagging behind the change in mixing layer height, rather than a synchronous change. Furthermore, recent research found that the maximum rate change of ultrafine particle concentrations observed in the near-surface layer was always preceded by breakdown of the nocturnal inversion and enhancement of vertical mixing [66].

### 3.3. Diurnal Variations of Particle Number Concentration in the Four Seasons

**Figure 4:**
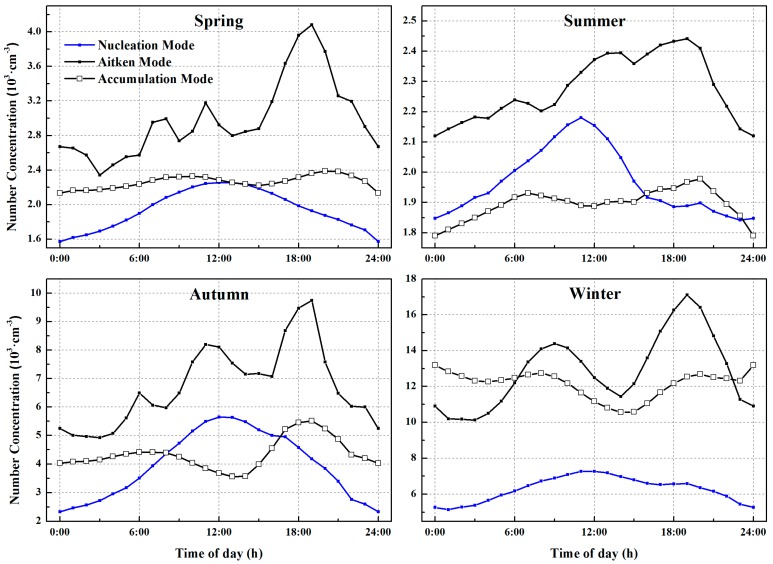
Diurnal variations in averaged particle number concentrations in the nucleation, Aitken, and accumulation modes during the four seasons.

The diurnal patterns of particle number concentrations in the different modes averaged over the four seasons are illustrated in Figure 4. In this study, spring refers to March, April, and May; summer consists of the months of June, July, and August; autumn includes September, October, and November; and winter refers to December, January, and February. The two-year experimental data covered each season exactly twice. The particle number concentrations in the nucleation mode (blue lines) increased in the morning and declined in the afternoon throughout the year with a parabolic vertex around 12:00 local time (LT) (UTC + 8 h), probably caused by photochemical processes [67]. This pattern of new particulate formation events was apparent and the frequency of this event was relatively high in summer and autumn, possibly caused by adequate sunshine during these two seasons. The particles in Aitken mode (black lines with solid squares) could be demonstrated as a triple-peak distribution over the spring, summer, and autumn, as Aitken particulates increased obviously during the midday peak, morning traffic rush hour that commenced around 06:00 LT, and evening traffic rush hour that occurred around 17:00 LT. However, the particles in Aitken mode had a bimodal distribution in winter with no midday peak, which may be explained by a low growth rate of particles. References [68,69] reported that the growth rate in winter had been calculated to be the lowest of the four seasons, leading to the conclusion that the newly formed particles or the aged particles in the nucleation mode cannot grow into particles in the Aitken mode during the daytime. The morning peak in the Aitken mode was affected mainly by dense traffic density combined with lower ambient temperature and lower mixing layer height. The night peak was linked tightly to the evening traffic rush hour, which usually lasts longer than the morning traffic rush hour does, leading to higher number concentrations in the Aitken mode, and the decreasing mixing layer height, which may lower simultaneously the intensity of vertical dilution and mixing of particles during the night [10]. Moreover, a higher mixing layer height leads to stronger dispersion in contrast to that in other seasons, which results in lower peak values during summer. Furthermore, the duration of the increase in particle number concentrations in winter was longer than it was during other seasons, which could probably be explained by the low mixing layer height and low temperature accelerating the condensation of exhaust gases and decelerating the diffusion of them [70]. In addition, particles in the accumulation mode (black lines with hollow squares) had a weaker relationship with traffic rush hour during the four seasons, which means that the traffic-related emissions were found predominantly in the Aitken mode in Wuhan, as they were in other urban cities.

### 3.4. A Case Analysis of a Nucleation Burst Event in Winter

**Figure 5:**
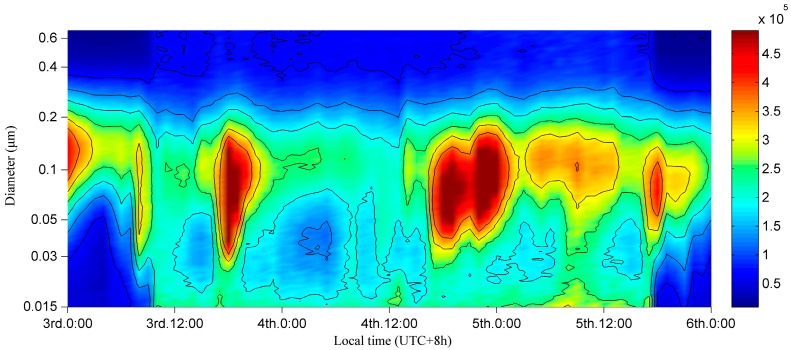
Contour plot of the size distribution of number concentration, $\text{d}N/\mathrm{dlog}D_{p}$ in cm^−3^ on three consecutive winter days from 3 January to 5 January 2014.

As demonstrated via a contour plot of size distributions in number concentration, Figure 5 displays an integral process, comprising a nucleation burst event and scavenging event, of particle number concentrations from data observed in winter from 3 January to 5 January in 2014. This three-day phenomenon is of vital significance to revelations about the durability of particulate pollution during winter in Wuhan. General overview of a two-year dataset demonstrated that durable ultrafine particle pollutions emerged four times and nine times during winter, respectively, from 2012 to 2013 and from 2013 to 2014. Each similar ultrafine particle pollution in winter lasted roughly from one day to one week. During this experimental period, the weather was partly cloudy with no precipitation.

**Figure 6:**
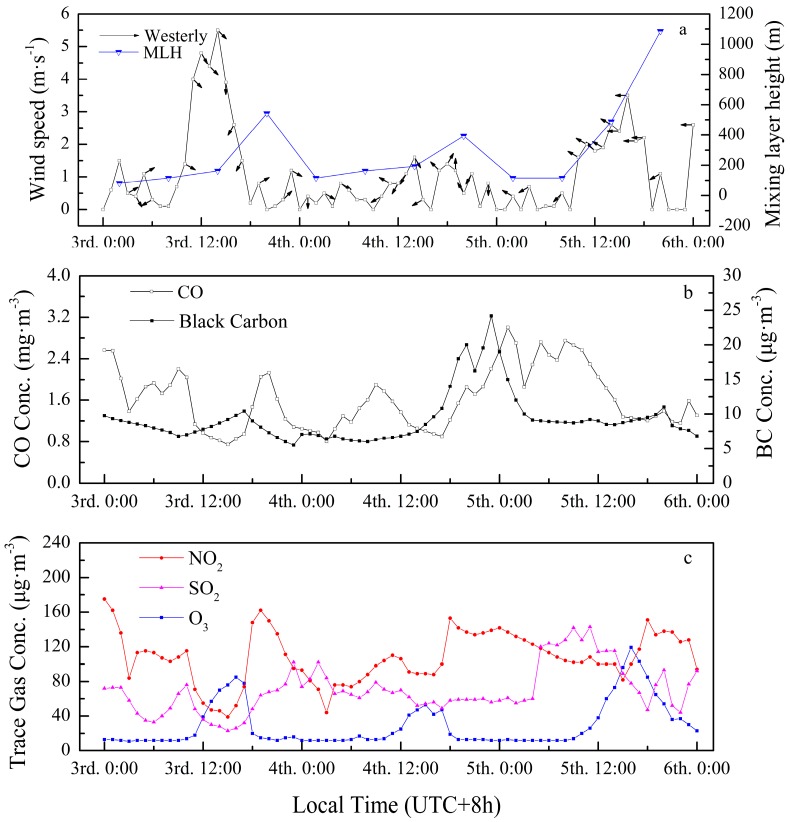
Concentration of trace gases averaged from three sites and black carbon with meteorological parameters on three consecutive winter days from 3 January to 5 January 2014: (**a**) hourly variations of wind speed and mixing layer height; (**b**) hourly variations in mass concentrations of CO and black carbon; (**c**) hourly variations in mass concentrations of NO_2_, SO_2_, and O_3_.

From a macro scale, it is obvious that the normalized number concentration of particles in the nucleation mode and partly in the Aitken mode sharply increased from approximately 5 × 10^4^ cm^−3^ to 2 × 10^5^ cm^−3^ around 10:00 LT on 3 January and rapidly decreased around 17:00 LT on 5 January, seemingly corresponding to the wind curves shown in Figure 6a. The fact that the variation of wind was earlier than the saltation of particles in nucleation mode, which corresponded tightly with the wind direction, cannot be ignored. The northwest wind began on 8:00 LT with an increase in mass concentrations of SO_2_, NO_2_, and particles ranging from 30 nm to 200 nm. These additional particles were consistent with the features of long-range transported aerosols described by [27] that import-particles mainly consist of accumulation mode particles, as nucleation mode particles have short lifetimes due to their condensation growth. In addition, NO_2_ is one of the primary reactants in photochemical processes, in which atomic oxygen and O_3_ have been produced [71,72]. Combined with the effect of exogenous SO_2_ oxidizing, a nucleation event emerged with nanoparticles growing into detectable particles by condensation or coagulation, proving that SO_2_ could be considered a crucial precursor for nucleation events in Wuhan, as it was in Nanjing [73]. The southeast wind began at 11:00 LT on 5 January and is considered the end of the lasting pollution and is accompanied by the elevation of mixing layer height, as shown in Figure 6a. This expansion of atmosphere may have broken the thermal inversion layer by strengthening the intensity of vertical dilution, leading to the diffusion of fine particles of pollution.

From the diurnal pattern of fine particles, periodic pollution existed in the afternoons and evenings, with increases in mass concentration of CO, NO_2_, SO_2_, and BC, as shown in Figure 6. As NO_2_ and CO are produced primarily by high temperature combustion in an urban atmosphere, traffic intensity and industrial activities should be responsible for this type of pollution. Although similar bimodal distributions of trace gases during the traffic rush hours occurred, such similarities were inconspicuous in the contour plot of the size distribution of number concentration in the three modes. A possible explanation for this is that vehicle exhaust emissions were not detectable synchronously under a series of complex chemical reactions or the particle number concentrations of traffic-related emissions were not on the same order of magnitude as the existing pollution. Based on Figure 6, industrial emissions were reflected mainly in the mass concentrations of NO_2_, SO_2_, and BC. Moreover, the O_3_ concentration, which is considered generally as a proxy for the intensity of photochemical processes, illustrated a unimodal distribution for its diurnal pattern, reaching its peak around 15:00 LT, which is later when compared to usual situations. Furthermore, since NO_2_ is a depleting substance in photochemical processes, its mass concentration showed a similar profile affected by traffic and industrial emissions simultaneously, but varied in the opposite direction to O_3_ most of the time. Last but not the least, although particles ranging from 20 nm to 100 nm tended to decrease several times, it never came to fruition because the meteorological conditions were quite stable on 4 January. It could be inferred that the repeated increase in ultrafine particles was attributed to lately generated SO_2_ via industrial activities, contributing to the “repeated, short-lived” nucleation events, which contrast with the “short-lived” events by [74,75]. In conclusion, exogenous pollutants were the fuse for this burst of ultrafine particles, as the stagnant atmosphere caused by a thermal inversion layer tremendously impeded the diffusion of particles combined with dense industrial activities and traffic emissions, and clean northwest wind broke the stability of the lower atmosphere, accelerating the dispersion of particles with the expansion of the mixing layer.

## 4. Conclusions

Two-year continuous measurements of particle number concentration and meteorological data and other parameters, including mass concentration of SO_2_, NO_2_, CO, O_3_, and BC, were conducted in the city of Wuhan from June 2012 to May 2014. During the experimental period, the average number concentrations of submicron particles in the nucleation (15–30 nm), Aitken (30–100 nm), and accumulation (100–661 nm) modes reached 4923 cm^−3^, 12,193 cm^−3^ and 4801 cm^−3^, respectively. Compared to cities around the world, the particle number concentrations in the three modes in Wuhan were relatively high, directly influenced by the specific level of industry and energy structure.

Pearson coefficients showed that there were distinct differences among the correlation of particle number concentrations in the three modes with precipitation, wind speed, RH, AP, temperature, and mixing layer height. Precipitation, AP, and temperature played a vital role in shaping the number concentration of particles in the three modes, whereas RH and mixing layer height did not appear to have synchronous change with particle number concentrations in Wuhan. Wind speed had a significant negative effect on particle number concentrations in the nucleation and accumulation modes.

According to the diurnal variations of particle number concentration in four seasons, particles in the nucleation mode demonstrated a unimodal distribution which reached their peak around noon, with more active nucleation events in summer and autumn. The particles in the Aitken mode showed a triple-peak distribution in spring, summer, and autumn, but a bimodal distribution in winter, possibly caused by a lower rate of new particle formation due to less condensable vapours and solar radiation. The two peaks of Aitken and accumulation mode particles during the morning and evening corresponded to the traffic rush hours, likely due to traffic exhaust emissions.

A three-day case analysis was conducted to explore the key factors leading to the durability of high particle concentrations during winter, which contained a complete process comprising a nucleation burst event and a scavenging event. This burst of ultrafine particles resulted from exogenous pollutants, with the thermal inversion layer tremendously impeding the diffusion of particles. These stagnant atmosphere conditions were a hotbed of “repeated, short-lived” nucleation events, combined with dense industrial activities and traffic emissions. The clean northwest wind finally broke the stability of the lower atmosphere, promoting the dispersion of particles with the expansion of the mixing layer.

This paper presents a systematic analysis of fine particles in Wuhan, the first such study performed in the central plains of China, and their relationships with climate, meteorological parameters, atmospheric factors, trace gases, and industry levels. To enhance these results, additional factors should be taken into consideration, such as traffic density, detailed industrial emissions, and quantified health effects. In addition, the research into the mechanism of nucleation event and growth ratio about ultrafine particles will be carried out in the next stage. Moreover, this study offers reasonable estimations of particle impacts and provides guidance for policy making and emission control strategies in Chinese cities with heavy industry.

## Figures and Tables

**Table 1:** Overview of comparisons of particle number concentrations in different locations of the world.

Number Concentration (cm^−3^)	Nucleation Mode	Aitken Mode	Accumulation Mode	Total Number	References
Diameter range	3–25 nm	25–90 nm	90–500 nm	3–500 nm	
Hyytiälä, 3 years	480	1010	620	2110	[50]
Diameter range		10–100 nm	100–500 nm	3–500 nm	
Alkmaar, half year		18,300	2120	25,800	[51]
Erfurt, half year		17,700	2270	25,900	[51]
Diameter range	3–10 nm	10–100 nm	100–2000 nm	3–2000 nm	
Atlanta, 1 year	5564	13,482	1690	20,736	[52]
Diameter range	3–20 nm	20–100 nm	100–800 nm	3–800 nm	
Leipzig, 4 years	9850	9413	2107	21,377	[25]
Diameter range	8–30 nm	20–100 nm	90–400 nm		
Helsinki, 6 years	7100	6320	960		[23]
Diameter range	3–20 nm	20–100 nm	100–1000 nm	3–2500 nm	
Pittsburgh, 1 year	9700	10,100	2188	21,988	[26]
Diameter range	3–20 nm	20–100 nm	100–1000nm	3–10,000 nm	
Beijing, 2 years	9000	15,900	7800	32,800	[10]
Diameter range	13–20 nm	20–100 nm	100–800 nm	13–800 nm	
Barcelona, 1 year	2340	11,820	2630	16,890	[53]
Diameter range	10–30 nm	30–300 nm	300–800 nm		
Augsburg, 2 years	3700	7600	150	11,450	[54]
Diameter range				10–289 nm	
Seoul, 8 years				17,811	[55]
Diameter range	10–25 nm	25–100 nm	100–1000 nm	10–1000 nm	
Lanzhou, 1 year	1799	16,083	4964	22,846	[43]
Diameter range	15–30 nm	30–100 nm	100–661 nm	15–661 nm	
Wuhan, 2 years	4923	12,193	4801	21,917	This study

**Table 2:** Pearson coefficients between particle number concentrations in the three modes and average meteorological parameters. The * means significance of *p* < 0.05, the ** means significance of *p* < 0.01.

Pearson Coefficients	Nucleation Mode	Aitken Mode	Accumulation Mode
Precipitation	−0.505 *	−0.477 *	−0.537 **
Wind speed	−0.434 *	−0.390	−0.408 *
Relative Humidity	−0.186	−0.262	−0.233
Atmospheric Pressure	0.547 **	0.544 **	0.579 **
Temperature	−0.567 **	−0.515 *	−0.563 **
Mixing layer height	−0.258	−0.159	−0.226
